# Prediction of Personalised Hypertension Using Machine Learning in Indonesian Population

**DOI:** 10.1007/s10916-025-02253-5

**Published:** 2025-10-13

**Authors:** Edo Septian, Muhammad Rizal Khaefi, Achmad Athoillah, Dewi Nur Aisyah, Muhammad Hardhantyo, Fauziah Mauly Rahman, Logan Manikam

**Affiliations:** 1 Digital Transformation Office, Ministry of Health Republic of Indonesia, Jalan H.R Rasuna Said Blok X5 Kav. 4-9, South, 12950 Jakarta, Indonesia; 2 Department of Epidemiology and Public Health, Institute of Epidemiology and Health Care, University College London, 1-19 Torrington Place, WC1E 7HB London, UK; 3 Aceso Global Health Consultants Pte Limited, 10 Anson Road, #23-08A, 079903 Singapore, Singapore; 4 Department of Public Health, Monash University Indonesia, Green Office Park BSD City, 15345 Tangerang, Indonesia; 5 The World Bank Group, Washington, DC United States of America; 6 Center for Health Policy and Management, Faculty of Medicine, Public Health and Nursing, Universitas Gadjah Mada, Yogyakarta, Indonesia; 7 Faculty of Health Science, Universitas Respati Yogyakarta, Yogyakarta, Indonesia; 8 School of Computer Science, Faculty of Engineering, University of Sydney, Camperdown, NSW Australia

**Keywords:** Hypertension prediction, Machine learning, Non-communicable diseases (NCDs), Risk assessment

## Abstract

This study aims to enhance individual hypertension risk prediction in Indonesia using machine learning (ML) models. The research investigates the predictive accuracy of models with and without incorporating personal hypertension history, seeking to understand how data limitations impact model performance in a low-resource setting. Data from the SATUSEHAT IndonesiaKu (ASIK) system were preprocessed and filtered to create a dataset of 9.58 million adult health records. Two primary model variations were compared: Model A (incorporating patient history) and Model B (excluding patient history). We evaluated the model using five algorithms: XGBoost, LightGBM, CatBoost, Logistic Regression, and Random Forest. Model performance was assessed using the Area Under the Curve (AUC), sensitivity, and specificity metrics. Model A achieved superior predictive accuracy (AUC = 0.85) compared to Model B (AUC = 0.78). To mitigate potential bias, Model B was selected for further in-depth development. Evaluation of model B reveals that XGBoost and LightGBM algorithm achieved the highest performance (AUC 0.78) and LightGBM emerged as the best algorithm based on its performance. SHAP analysis was conducted and identified key predictors such as age, family history of hypertension, body weight, and waist circumference. This study finds that while a patient’s personal history of hypertension significantly enhances predictive accuracy, robust ML models can effectively predict hypertension risk using other accessible demographic, clinical, and lifestyle features. Model B offers a valuable and generalizable approach for broader risk screening, particularly where patient history may be unavailable or unreliable, while also providing insights into key modifiable and non-modifiable determinants of hypertension.

**Supplementary Information:**

The online version contains supplementary material available at 10.1007/s10916-025-02253-5.

## Background

Non-communicable diseases (NCDs) are currently the leading cause of death and disability worldwide, including in Indonesia [1, 2]. Hypertension is one of the five major NCDs [3], among these, hypertension contributes to many of the other NCDs and is often termed the “silent killer” [4]. Its asymptomatic nature often leaves individuals unaware of their condition until it escalates or turns into one of the other, symptomatic NCDs, resulting in complications such as stroke, coronary heart disease, and kidney failure.

Hypertension poses significant health concern in Indonesia, with a prevalence of approximately 25.8% [5]. However, only 8% of hypertension cases are detected by healthcare providers, and just 26.97% of those affected are aware of their condition. Reasons for low detection rates and low awareness rates in Indonesia include limited access to healthcare, particularly in rural areas, and the uneven distribution of services under the BPJS national health insurance scheme, which impacts routine monitoring availability [6, 7]. Furthermore, not all households own blood pressure monitoring devices, especially those that are validated and capable of providing consistent accuracy—essential for reliable health monitoring [1, 8]. While basic devices might be affordable, access to more precise, validated tools remains limited due to financial barriers and supply challenges. These issues are compounded by financial constraints and cultural attitudes that do not prioritize regular health monitoring, often leading to reliance on unvalidated devices, leading to unreliable diagnosis and monitoring [9].

While these challenges underscore the need for better healthcare access and awareness, technological innovation offers promising solutions. Internet connectivity in Indonesia has grown significantly over the past decade and mobile phone ownership is high, reaching 94.56% in urban areas and 89.34% in rural areas [10, 11] provides a platform for digital health solutions. Recent advancements in artificial intelligence (AI) and machine learning (ML) have shown potential in enhancing hypertension detection and management. AI-based techniques, including non-invasive blood pressure monitoring and risk assessment models, have been developed, demonstrating significant potential. For example, [12] developed a Random Forest machine learning algorithm using single-lead ECGs from 1,254 subjects, achieving 81% accuracy to distinguish between hypertensive and non-hypertensive individuals, with key features including age, BMI, and various ECG-derived measurements [12]. Similarly, innovative approaches by Yan et al. (2022) and Yuxue et al. (2024) have utilized multi-feature fusion methods and neural networks to improve blood pressure prediction accuracy [13, 14].

Integrating machine learning into hypertension management offers significant promise for advancing early detection and personalized treatment strategies. Machine learning models, including Random Forest and Gradient Boosting, have demonstrated superior accuracy in predicting hypertension compared to traditional methods [15]. Moreover, ML-based approaches have enabled the use of polygenic risk scores to refine blood pressure predictions, highlighting the importance of genetic factors in understanding hypertension [16].

Indonesia has been undergoing a significant digital transformation in healthcare aimed to integrate data unification and digitize health information across the country. SATUSEHAT platform is an ecosystem developed by the Ministry of Health of the Republic of Indonesia [17], incorporating multiple tools and applications to support healthcare delivery and public health efforts, enabling better data-driven decision-making. Digital health platforms like SATUSEHAT present a promising solution by providing tools for self-screening and early detection of hypertension risk factors, bridging the gap between the need for routine monitoring and healthcare accessibility [17].

Previous studies that explore machine learning for hypertension prediction in Indonesia use a valuable but older dataset conducted in 2014–2015 and the other one used a limited sample in one province [18, 19]. This study introduces critical novelty by utilizing national level data collected in 2023 from the country’s first integrated digital health platform. This study aims to leverage data from SATUSEHAT to develop personalized predictive models that assess the risk of hypertension in the Indonesian population, thereby supporting SATUSEHAT’s mission to enhance preventive health services and promote early detection of non-communicable diseases. By utilizing data from a variety of sources, clinical, lifestyle, and socio-demographic, predictive algorithms within these platforms can provide personalized risk assessments, facilitating early interventions and reducing the burden of hypertension-related complications.

## Methods

### Data Collection

This study employed a cross-sectional design to analyze de-identified health records from the SATUSEHAT IndonesiaKu (ASIK) application. Data for each individual was captured at a single encounter to identify demographic, clinical, and lifestyle factors associated with their hypertension status at the time of measurement. The goal was to use these associations to develop and validate a machine learning model for predicting hypertension risk.

Clinical trial number: not applicable. This study utilized de-identified data obtained from the SATUSEHAT IndonesiaKu (ASIK) application, obviating the need for direct patient consent. ASIK is an integral part of the SATUSEHAT National Health Information System, developed by the MoH of Indonesia. This mobile application is designed for health care workers in primary care to capture patient data, report healthcare services delivered outside community health centers (Puskesmas), input routine screening health information, and monitor patients conditions [20, 21].

Each record in this study corresponded to a single health facility encounter documented in the ASIKapplication. To create an individual-level dataset analysis, these records were systematically processed using valid citizen national identification numbers, which allowed for the linkage of multiple health facility visits to unique individuals. For each individual, the most recent clinical measurements were prioritized. This approach was adopted to ensure the currency of data and to mitigate potential biases arising from temporal variability in hypertension status and associated risk factors, thereby enhancing the reliability of our predictive models.

The initial dataset comprised 21,455,311 medical records from patients aged 18–90 years, collected throughout 2023 across all 38 provinces in Indonesia. This scope aligns with our study’s objective of assessing adult hypertension risk. To manage this large-scale and complex dataset, we utilized Google BigQuery, a cloud-based data warehouse solution. This platform enabled data processing and management while ensuring adherence to the stringent performance and security standard requisite for handling sensitive healthcare data [22].

### Target Variable Definition

We use classification of hypertension based on blood pressure measurements recorded by healthcare professionals at health facilities. This approach ensured that our classification of hypertensive individuals was based on professional medical assessments, adhering to the standardized diagnostic criteria established by healthcare providers in clinical settings. The clinical guideline for management of adult hypertension in Indonesia used the following classification; normal (120–129/80–84 mmHg), prehypertension (130–139/85–89 mmHg), and hypertension (≥ 140/90 mmHg) [23]. For our binary classification model, individuals with blood pressure readings in the hypertension category were classified as hypertensive cases, while those with readings in the normal or prehypertension categories were classified as non-hypertensive.

### Model Design

To evaluate the specific predictive contribution of a patient’s known history of hypertension, we developed two distinct model variations: Model A, which included the patient’s history of hypertension as a predictor value, and Model B, which excluded this feature. This comparative approach enabled a quantitative assessment of this variable’s impact on predictive accuracy. It also addressed the practical consideration of varying patient awareness, accommodating individuals who self-report previous high blood pressure readings as well as those with no prior knowledge of their hypertension status. Crucially, this dual-model approach was established to ensure Model B could serve as a valid predictive tool for initial screening, thereby avoiding the circular logic of using a known diagnosis to predict the very same condition.

Information regarding a patient’s history of hypertension was obtained via a self-report during routine interviews conducted by healthcare workers at patient registration or screening. This was collected using structured, recall-based questionnaires that also gathered other lifestyle and medical history details. While the primary source was self-report, these data were often cross-referenced against prior diagnoses made by healthcare professionals and documented within the ASIK system. Consequently, the ‘patient history of hypertension’ variable often reflected a combination of patient self-awareness and formal clinical confirmation.

### Model Development

We developed predictive models for hypertension risk using the following research framework (Appendix 1). All data was collected by healthcare professionals during patient screenings at health facilities and entered into the ASIK application. Data collected from the mobile application is presented in Table 1.

**Table 1:** Patient screening variables from SATUSEHAT Indonesia-Ku(ASIK) mobile application

Feature	Definition	Measurement Tool
Clinical Variables		
Age (years)	The individual’s age Input in birth date (dd/mm/yyyy)	Recall questionnaire
Body Weight (kg)	The individual’s weight Input in kilograms	Weighing scale
Body Height (cm)	The individual’s height Input in centimeters	Height scale
Waist Circumference (cm)	Measurement of the individual’s abdominal circumference Input in centimeters	Body measuring tape
Lifestyle Variables		
Physical Activity	How often does the individual engage in physical activity? - Rarely (< 150 min/week) - Often (≥ 150 min/week)	Recall questionnaire
Sugar Consumption	Does the individual consume sweet foods or drinks frequently? - Excessive (≥ 4 tablespoons/day) - Normal (< 4 tablespoons/day)	Recall questionnaire
Salt Consumption	Does the individual consume salty foods frequently? - Excessive (≥ 1 teaspoon/day) - Normal (< 1 teaspoon/day)	Recall questionnaire
Oily Food Consumption	Does the individual consume oily foods frequently? - Excessive (≥ 5 tablespoons/day) - Normal (< 5 tablespoons/day)	Recall questionnaire
Medical History		
Diabetes Mellitus Diagnosis	Has the individual been diagnosed with diabetes mellitus by a doctor? - Yes/No/Unsure	Recall questionnaire
Family History of Hypertension	Does the individual’s family (father/mother/sibling) have a history of hypertension? - Yes/No/Unsure	Recall questionnaire
Patient History of Hypertension	Has the individual been diagnosed with hypertension by a doctor? - Yes/No/Unsure	Recall questionnaire

The development of our machine learning models, including training and subsequent analysis, was performed using Google’s Vertex AI platform [24]. This environment provided the scalable infrastructure and essential computational resources required for these tasks. The model development process comprised the following key steps:

#### Data Preprocessing

Data preprocessing is a critical step for enhancing the performance and reliability of machine learning models. Our methodology involves identifying and removing outliers, handling missing values, and performing feature transformations.

- Outlier Handling:

- To manage outliers, clinically appropriate value ranges were established for key anthropometric variables; body weight, height, waist circumference, and body mass index (BMI). This was done in collaboration with subject matter experts to ensure that the data cleaning was grounded in medically relevant criteria. Records containing value outside these expert-defined ranges were excluded.

b) Missing Data Management:

- Records with incomplete data for any required feature were removed using a complete case analysis approach which is the*dropna* method. We opted against imputation techniques, such as mean or median substitution, to avoid introducing estimated values that could potentially compromise the clinical validity of our analyses in this medical hypertension study. This decision was supported by the substantial size of our dataset; after exclusion, 9,580,646 complete records remained, providing sufficient statistical power for robust analysis. This approach aligns with literature suggesting that when large sample sizes are retained, the loss of precision from complete case analysis is often minimal compared to the potential bias introduced by imputation, particularly when data may not be missing completely at random in medical context [25, 26].

c) Feature Transformation:

- Categorical features were converted to ‘category’ data type and then encoded into binary (0/1) variables using OneHotEncoder to facilitate processing by machine learning algorithms. Numerical features were used in their original scale. Normalization was deemed unnecessary, as LightGBM model, being a tree-based algorithm, inherently handles with different scales well effectively by making decisions based on values splitting points rather than absolute numerical values [27].

The features included in the model were.

- Categorical: physical activity level, sugar consumption habits, salt consumption habits, oily food consumption frequency, family history of hypertension, patient history of hypertension and history of diabetes mellitus.
- Numerical: age (years), body weight (kg), height (cm), and waist circumference (cm).

BMI was intentionally excluded as a direct feature to prevent redundancy, given that its component variables (weight and height) were already included. This also simplifies data input for potential end-users of the model.

**Fig. 1:**
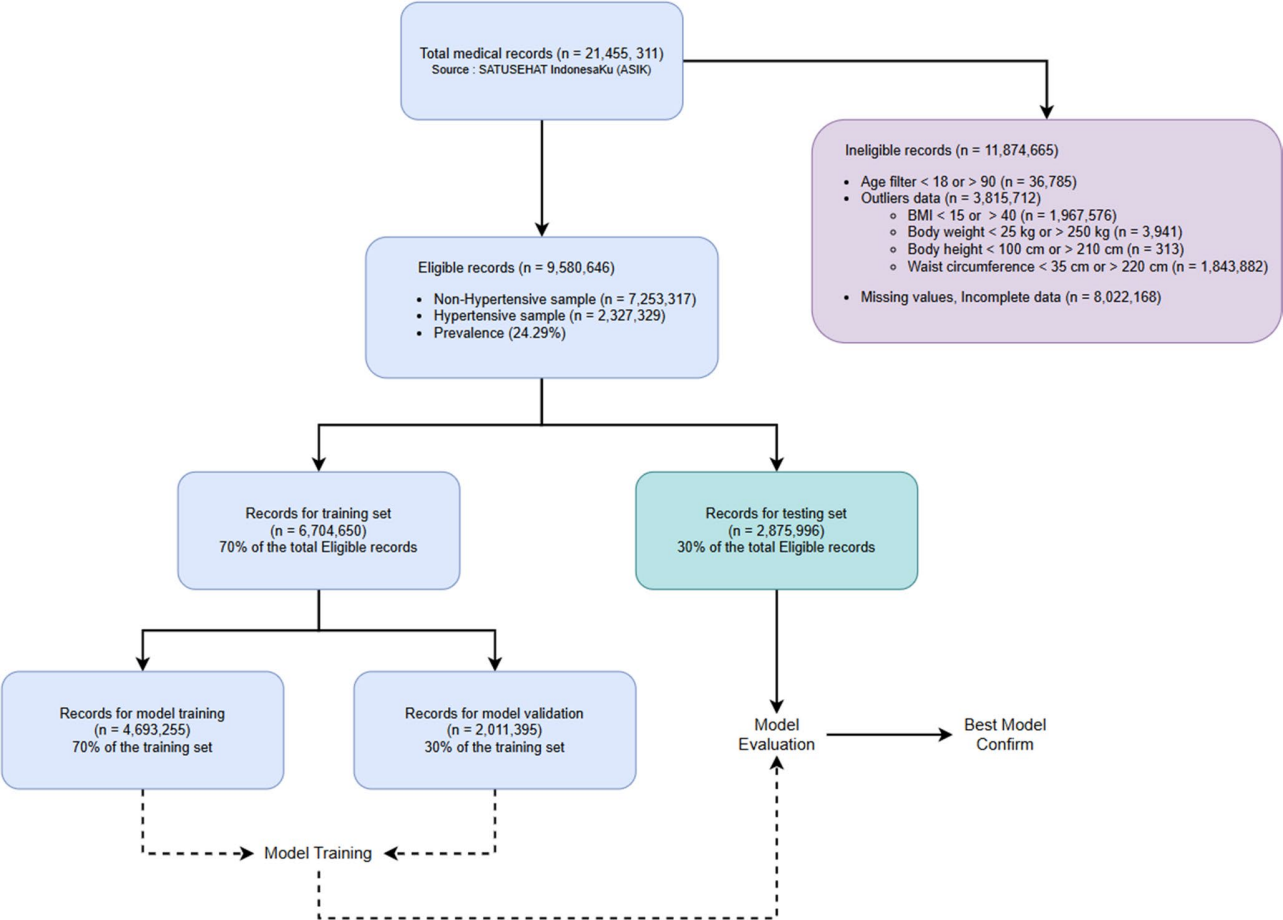
Data Cleaning

The preprocessing step systematically refined the dataset. As illustrated in Fig 1, the initial dataset sourced from ASIK contained 21,455,311 medical records. A total of 11,874,665 records were excluded based on the following criteria:

- individuals aged under 18 or over 90 years (*n* = 36,785).
- records with extreme outlier values in anthropometric data, as defined by clinical experts (*n* = 3,815,712). These included.
  - BMI < 15 or > 40 (*n* = 1,967,576).
  - body weight < 25 kg or > 250 kg (*n* = 3.941).
  - height < 100 cm or > 210 cm (*n* = 313).
  - waist circumference < 35 cm or > 220 cm (*n* = 1,843,882).
- records with missing data for any of the required features (*n* = 8,022,168).

Following these exclusions, a final dataset of 9,580,646 records were available for model development. This refined dataset served as the foundation for our subsequent analysis and model development.

#### Dataset Splitting

To ensure robust model development and evaluation while mitigating risks of overfitting and data leakage, the eligible dataset of 9,580,646 records was systematically partitioned, (illustrated in Fig. 1). The initial division randomly allocated records into two primary sets; a training set comprising 6,704,650 records (70% of the total), designed exclusively for model development, and an independent testing set with 2,875,996 records (30% of the total), reserved strictly as out-of-sample data for final model performance assessment.

For model development purposes, the training set was further split into a training subset of 4,693,255 records (70% of the training set) used for actual model training process and a validation subset of 2,011,395 records (30% of the primary training set) for hyperparameter tuning and internal validation. The testing set of 2,875,996 records remained entirely sequestered until the final evaluation phase. This strict separation ensured an unbiased assessment of the finalized model’s generalization capabilities on unseen data. This structured partitioning strategy provided a distinct dataset for model training, optimization, and a rigorous final performance evaluation.

#### Model Training

We then experimented with various machine learning algorithms to find the optimal model for hypertension prediction, including XGBoost [27], LightGBM [28], CatBoost [29], Logistic Regression [30], and Random Forest [31], as shown in Table 2. Ensemble methods like XGBoost and LightGBM were chosen for their proven effectiveness in handling large, complex datasets and capturing the non-linear relationships often present in health data. Logistic Regression was included as a robust and interpretable baseline model to benchmark the performance of the more complex algorithms. Each algorithm was configured with specific hyperparameters and integrated into a scikit-learn pipeline to ensure consistent preprocessing across all models.

**Table 2:** Machine learning algorithms performed in this study

Algorithm	Description	Usage
XGBoost [27]	An efficient and scalable implementation of gradient boosting, known for high prediction accuracy and handling large datasets.	Used for its robustness in classification tasks and managing overfitting.
LightGBM [28]	A gradient boosting framework that is optimized for speed and efficiency, particularly suitable for handling large-scale data with lower memory usage.	Employed for faster training times and lower memory usage.
CatBoost [29]	A gradient boosting algorithm designed to handle categorical data effectively, providing high accuracy and faster prediction speed.	Chosen for its strength in handling categorical features without extensive preprocessing.
Logistic Regression [30]	A statistical model used for binary classification, estimating the probability of a binary outcome based on one or more predictor variables.	Served as a baseline model for comparing performance with complex algorithms.
Random Forest[31]	An ensemble learning method that constructs multiple decision trees and averages their predictions to enhance accuracy and prevent overfitting.	Used to provide reliable predictions and improve model stability.

#### Model Evaluation and Validation

The model development process involved training on the designated training subset, with the validation subset used for hyperparameter tuning and preliminary performance assessment.

To ensure robust model validation, we implemented Stratified K-Fold cross-validation with five folds. This method guarantees that each fold reflects the same class distribution as the original dataset. By preserving the ratio of hypertensive to non-hypertensive samples across all validation folds, Stratified K-Fold provides more reliable performance estimates than traditional cross-validation techniques. This approach helps to reduce evaluation bias and ensures that each fold contains approximately equal proportions of both class labels, resulting in more consistent performance metrics. To evaluate the model's performance, we use Area Under the Curve (AUC) [32], along with sensitivity and specificity [33]. Detailed information about this metrics is available on Table 3.

**Table 3:** Definitions and purposes of model evaluation metrics

Metric	Definition	Purpose
ROC AUC [32]	Measures the model’s ability to distinguish between positive (hypertensive) and negative (non-hypertensive) cases. A higher AUC indicates better discrimination.	To provide a single value summarizing the model’s performance across all classification thresholds.
Sensitivity (Recall) [33]	Proportion of true positive cases (actual hypertensive individuals) correctly identified by the model. Calculated as the ratio of true positives to the sum of true positives and false negatives.	To ensure that individuals at risk of hypertension are accurately flagged for further evaluation.
Specificity [33]	Proportion of true negative cases (actual non-hypertensive individuals) correctly identified by the model. Calculated as the ratio of true negatives to the sum of true negatives and false positives.	To minimize false positives and reduce unnecessary follow-up tests and healthcare costs.

To enhance the interpretability of our predictive model and to understand the underlying drivers of its predictions, we employed SHAP (SHapley Additive exPlanations) values. SHAP is a game theory-based approach that explains the output of any machine learning model by quantifying the contribution of each feature to an individual prediction [34–36]. Detailed information about SHAP can be found in Appendix 2.

By calculating and analyzing these SHAP values, we identified which factors most significantly influenced the model’s hypertension predictions. This provided valuable clinical insights that extend beyond aggregate predictive performance, helping to elucidate how specific input features affected the model’s decision-making process for individual instances.

## Results

### Population Characteristics

After gathering data through the SATUSEHAT IndonesiaKu (ASIK), we conducted an analysis of our dataset’s characteristics. Our analysis comprised 17,602,814 records that include clinical, lifestyle, and socio-demographic features. While this dataset has been cleansed of outliers, we have retained the null value or NaN or left blank values to provide insight into the incomplete data at the row level dataset.

Tables 4 and 5 illustrate the distribution of numerical and categorical variables, respectively, segmented by hypertension status. As detailed in Table 4, individuals with hypertension were, on average, approximately 10 years older (mean age 49.96 years compared to 39.82 years), had a higher body weight (60.82 kg versus 58.39 kg), and exhibited a larger waist circumference (83.48 cm compared to 80.42 cm) than their non-hypertensive counterparts.

**Table 4:**
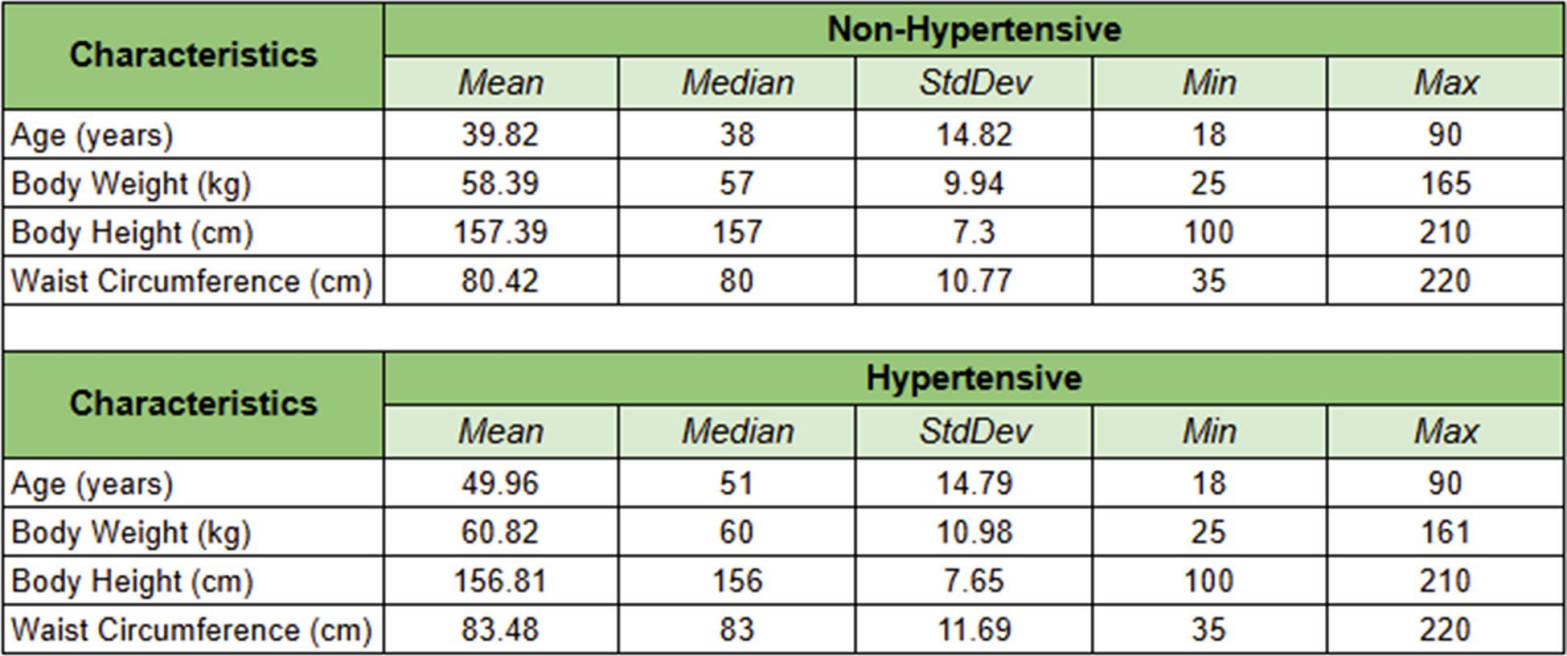
Statistical distribution of the study population

**Table 5:**
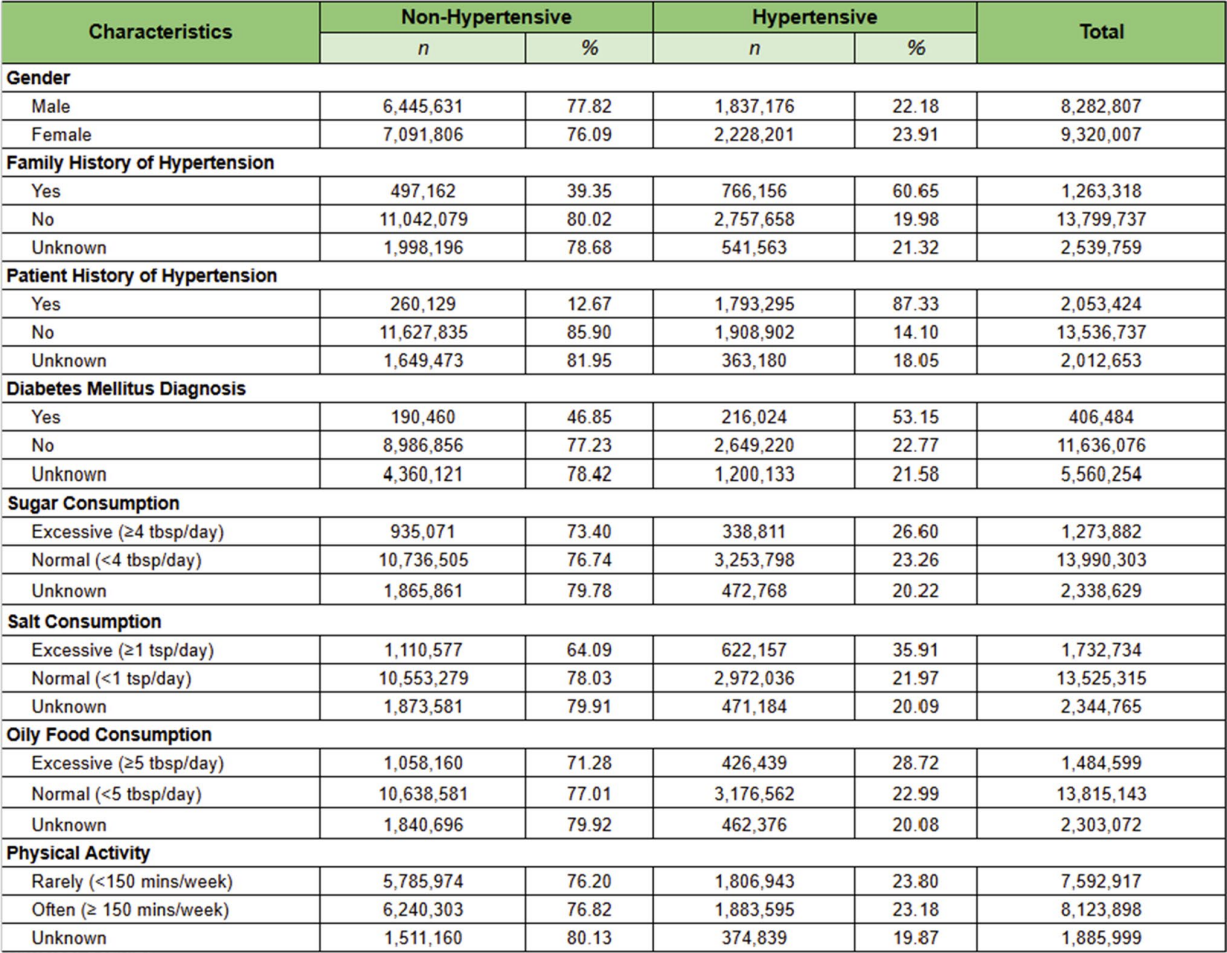
Detailed population characteristic

The categorical variables presented in Table 5 reveal several significant patterns. Notably, there is a marked imbalance in the ‘Patient History of Hypertension’ variable, with 87.33% of individuals with a history of hypertension classified as hypertensive in our dataset. This considerable imbalance raises concerns about potential bias in predictive modeling, as models may overly depend on this variable, which could limit their generalizability. Additionally, the prevalence of family history of hypertension (60.65% versus 39.35%), diabetes mellitus diagnosis (53.15% versus 46.85%), and excessive salt consumption (35.91% versus 64.09%) all demonstrated substantial differences between hypertensive and non-hypertensive groups.

### Model Performance

After developing and training the two model variations (Model A, which includes patient history of hypertension, and Model B, which excludes it) using the machine learning algorithms described in the Methods section, we evaluated their performance on an independent testing set of 2,875,996 records. The models were assessed based on their ability to predict hypertension risk accurately, using Area Under the Curve (AUC) as the primary metric, along with sensitivity and specificity. These metrics provide a comprehensive view of each model’s predictive power, balancing the ability to correctly identify both hypertensive and non-hypertensive individuals. The results below demonstrate the comparative performance of the two models, highlighting the impact of including or excluding personal hypertension history on predictive accuracy.

- Model Including Patient History of Hypertension (Model A)

- This model demonstrated superior performance with an AUC of 0.85, sensitivity of 0.69, and specificity of 0.85. The inclusion of a patient's personal history of hypertension appeared to enhance the model's predictive accuracy, suggesting a notable ability to identify individuals at risk of hypertension. These values suggest a well-balanced model performance, with particularly strong specificity in ruling out non-hypertensive cases.

b. Model Excluding Patient History of Hypertension (Model B)

- This model, excluding hypertension history of patients as an input variable, achieved a slightly lower AUC of 0.78, sensitivity of 0.71, and specificity of 0.69. While the model is still reasonably effective, it demonstrates a lower predictive performance compared to Model A. These values reflect moderate model performance in identifying hypertensive individuals, with a balanced approach between sensitivity and specificity. Although slightly less accurate than the model with hypertension history of patients, it still offers valuable insights for initial risk screening, particularly in scenarios where patient history data is unavailable.

**Fig. 2:**
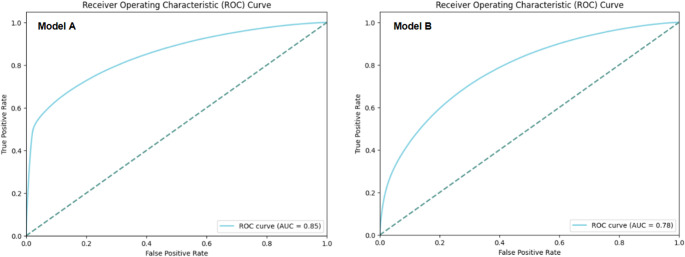
Comparison of ROC curves for hypertension prediction models with and without patient history of hypertension

To visually represent the performance difference between the two models, Fig. 2 displays the Receiver Operating Characteristic (ROC) curves for both Model A and Model B.

As shown in Fig. 2 Comparison of ROC Curves for Hypertension Prediction Models With and Without Patient History of Hypertension, the ROC curve for Model A demonstrates a larger area under the curve compared to Model B, visually confirming the superior discriminative power of Model A. To facilitate a direct comparison, Table 6 summarizes the key metrics for both Model A and Model B.

**Table 6:** Summarizes the performance metrics for both models

Metric	Model A	Model B
ROC AUC	0.85 (strong discriminatory power)	0.78 (reasonable performance)
Sensitivity (Recall)	0.69	0.71
Specificity	**0.85**	**0.69**

Despite Model A’s good performance, we chose to focus on Model B for further development. This choice directly aligns with the primary goal of developing a model for true *prediction* of risk in potentially undiagnosed individuals, rather than simple *confirmation* of a known condition. This decision was made to also mitigate the potential bias from the highly imbalanced ‘Patient History of Hypertension’ variable (87.33% association with the target variable) and to develop a more generalizable model. By excluding the Patient History of Hypertension variable, Model B achieves an AUC of 0.78 and enables a more thorough evaluation of other risk factors. This approach enhances the model’s applicability in situations where patient history may be unavailable or unreliable. Model B also achieves a higher recall score of 0.71 compared to model A, and this is important for screening purposes by minimizing the risk of missed hypertension cases.

### Algorithm Comparison

**Fig. 3:**
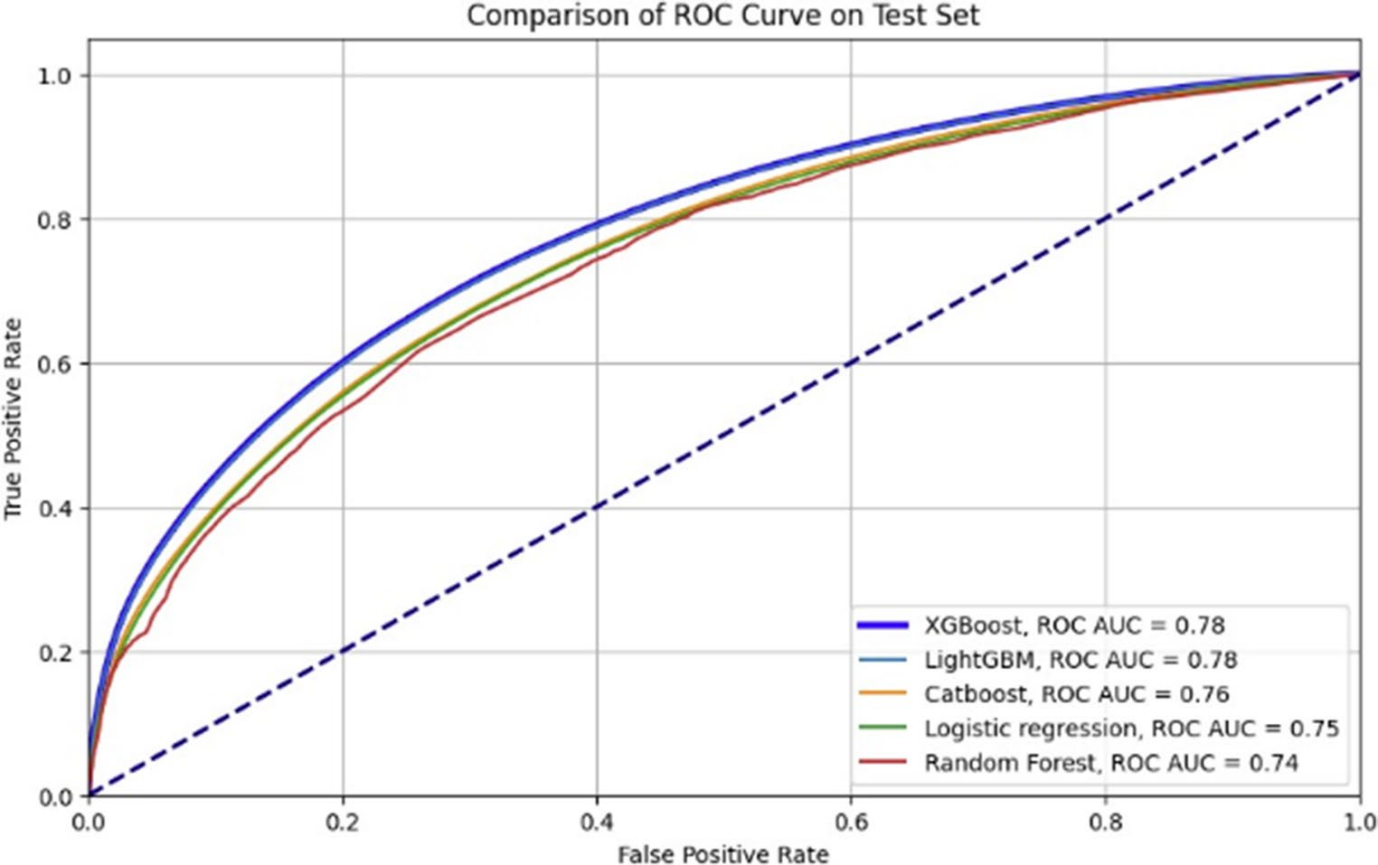
Comparison of algorithms performance

To gain deeper insights into the performance of various machine learning algorithms in Model B, we conducted a comparison of several approaches, including XGBoost, LightGBM, CatBoost, Logistic Regression, and Random Forest. As shown in Fig. 3, both XGBoost and LightGBM achieved the highest performance, with identical AUC scores of 0.78, followed closely by CatBoost (AUC = 0.76), Logistic Regression (AUC = 0.75), and Random Forest (AUC = 0.74).

Our findings underscore the effectiveness of ensemble methods, particularly XGBoost and LightGBM, in predicting hypertension risk. These gradient boosting frameworks demonstrated superior predictive capabilities compared to traditional algorithms like Logistic Regression. The enhanced performance of these ensemble methods can be attributed to their ability to capture complex, non-linear relationships between various risk factors and hypertension outcomes, which is especially valuable in the multifactorial context of cardiovascular disease prediction.

While both XGBoost and LightGBM demonstrated comparable predictive power, we ultimately chose LightGBM as our preferred algorithm due to its superior computational efficiency and smaller memory. An important factor in this decision was deployment compatibility; LightGBM integrates seamlessly within Alpine Docker environments, while XGBoost presented compatibility issues with these security-optimized containers. In accordance with our security team’s recommendations, we designed our implementation architecture to utilize Alpine Docker images *(FROM alpine:3.20*), which offer enhanced protection against security vulnerabilities compared to other alternatives.

### Model Testing and Stability Analysis

To evaluate the robustness and real-world applicability of Model B (excluding patient history of hypertension), we conducted extensive testing using data collected from January to June 2024. This longitudinal analysis allowed us to assess the model’s performance stability over time and across varying data volumes. Table 7 presents the detailed testing results for Model B across these six months:

**Table 7:** Model B testing results with 2024 data

Month	Testing data (million)	AUC	Sensitivity	Specificity
January	1.3	0.77	0.72	0.67
February	1.5	0.77	0.72	0.68
March	1.6	0.78	0.72	0.68
April	1.5	0.77	0.70	0.69
May	2.1	0.77	0.70	0.69
June	1.9	0.77	0.70	0.69

As evident from Table 7, Model B demonstrated consistent performance throughout the testing period. The AUC values remained stable, ranging from 0.77 to 0.78, indicating reliable predictive power. Sensitivity showed slight variations between 0.70 and 0.72, while specificity ranged from 0.67 to 0.69. Notably, the model maintained this consistent performance despite variations in monthly data volume, from 1.3 million records in January to 1.9 million in June. This stability across different sample sizes underscores the model’s scalability and robustness in handling large-scale, real-world data inputs.

### Feature Importance Analysis Using SHAP

**Fig. 4:**
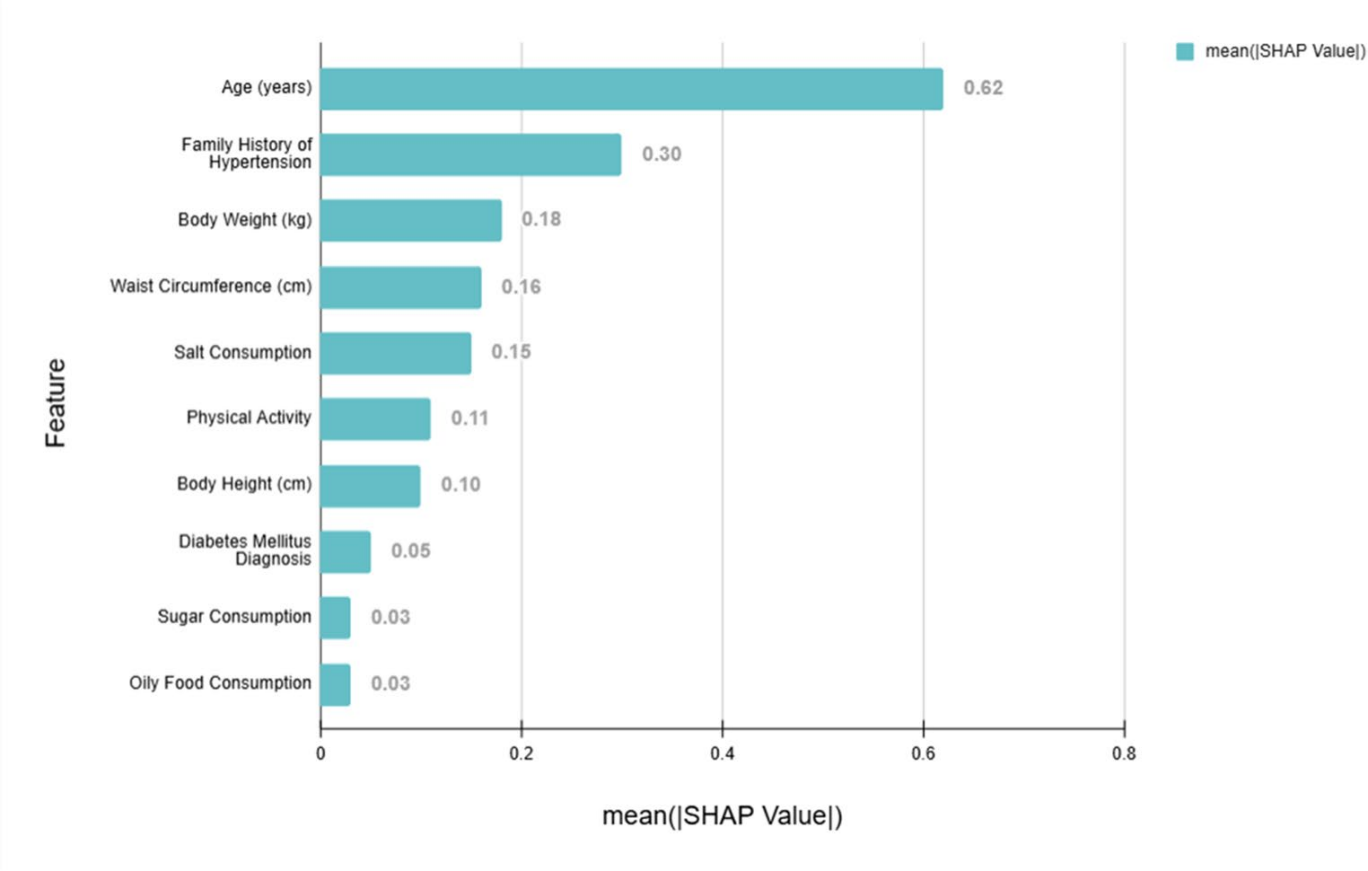
Feature importance analysis for model B (without self hypertension history)

The predictive models developed in this study utilized a comprehensive set of clinical, lifestyle, and socio-demographic features to assess the risk of hypertension. We performed an analysis of feature importance using SHAP (SHapley Additive exPlanations) values to identify the variables contributing most significantly to the models’ predictive accuracy. Utilizing the LightGBM algorithm, a tree-based model, which offers built-in support for efficient SHAP value computation, we generated these values. As shown in Fig. 4, our feature importance analysis for Model B (excluding patient history of hypertension) illustrates the relative contributions of each variable to the prediction outcomes.

#### Top Contributing Features

1. Age: Age emerged as the most important predictor in Model B, with a SHAP value of 0.62. This indicates that age is critical in assessing hypertension risk, especially in the absence of personal history data.
2. Family History of Hypertension: With a SHAP value of 0.30, family history was the next most influential factor, underscoring its role as a non-modifiable risk factor in hypertension prediction.
3. Other Clinical Variables: Variables like body weight (SHAP value: 0.18) and waist circumference (SHAP value: 0.16) also ranked highly, reflecting the strong association between body weight and abdominal fat distribution on hypertension risk.

#### Lifestyle Factors

1. Physical Activity: Higher levels of physical activity were strongly associated with a lower likelihood of having hypertension, with a SHAP value of 0.11.
2. Salt Consumption: Salt intake was a notable risk factor, with a SHAP value of 0.15, highlighting the importance of dietary salt control in managing blood pressure.

#### Less Contributing but Relevant Features

1. Sugar Consumption: Although it had a lower SHAP value (0.03), sugar consumption contributed to the model’s overall predictive capability.
2. Oily Food Consumption: Similarly, oily food intake was a minor factor with a SHAP value of 0.03, though it still played a part in the risk assessment.

This analysis of feature importance provides valuable insights into the key predictors of hypertension risk in our chosen model, highlighting both non-modifiable factors like age and family history, and modifiable lifestyle factors like physical activity and salt consumption. Notably, the model achieves good predictive power (AUC 0.78) without relying on the potentially biasing factor of personal hypertension history.

## Discussion

The results of this study demonstrate the effectiveness of machine learning (ML) algorithms in predicting hypertension risk, with clear implications for enhancing hypertension management in Indonesia. The two models developed, one with patient hypertension history and one without, showed that incorporating historical health data significantly improves predictive accuracy. These findings align with current research on the application of artificial intelligence (AI) and ML in healthcare, specifically in hypertension management, where personalized risk assessments are vital for early detection and intervention [12, 16]. These findings align with recent advancements in artificial intelligence (AI) and ML in healthcare, where the integration of personal and clinical data has proven essential for accurate disease prediction [37].

### Performance of Machine Learning Algorithms

Model A, which included personal hypertension history, exhibited superiorModel A, which included personal hypertension history, exhibited superior predictive accuracy (AUC = 0.85) compared to Model B (AUC = 0.78), confirming the importance of past medical history in risk assessments. In this study, XGBoost and LightGBM emerged as the best-performing algorithms, reflecting their robust capacity to handle large datasets and complex interactions between clinical and lifestyle factors [27, 28]. These findings are consistent with a recent systematic review by Estiko et al. (2024), which found that machine learning algorithms, including Random Forest, Gradient Boosting, and Extreme Gradient Boosting, consistently outperformed traditional methods in hypertension prediction, particularly when using easy-to-collect risk factors such as age, BMI, family history, and physical activity [15]. Similarly, Fang et al. (2023) demonstrated that a hybrid model combining KNN and LightGBM achieved a recall of over 92 percent in predicting five-year hypertension risk using comparable clinical features [38].

In contrast, Model B, which excluded patient hypertension history, still performed reasonably well, even with a lower AUC. This suggests that even in the absence of comprehensive historical data, machine learning models can still provide valuable risk screening using basic demographic and lifestyle information. This finding aligns with the results from Zhao et al. (2021), who demonstrated that machine learning models, particularly Random Forest, can predict hypertension risk using easy-to-collect data without the need for extensive clinical records [39]. Additionally, this finding also aligns with the broader landscape of hypertension prediction research. For instance, a recent systematic review highlighted that for hypertension risk prediction models, many of the successful models rely on conventional risk factors such as age, BMI, sex, which are typically readily available and easily collected [40]. Furthermore, when looking at the overall prediction interval of machine learning models from this meta analysis, the result showed a pooled c-statistic of 0.75 [40]. Model B performs significantly higher (AUC = 0.78) than this finding.

### Feature Importance in Hypertension Prediction

The feature importance analysis revealed that age was the most influential predictor, followed by family history of hypertension, body weight, and waist circumference. These findings are consistent with studies from [12, 16] and Chowdury et al. (2022), where age is among the top predictors of hypertension risk [12, 16, 40]. Additionally, waist circumference emerged as a key clinical variable, further underscoring the role of obesity and abdominal fat distribution in hypertension risk, as noted in previous research [39].

Lifestyle factors such as physical activity and salt consumption were identified as highly influential features in the model's prediction of hypertension risk. . This aligns with the literature, which consistently highlights the importance of exercise in managing hypertension and preventing cardiovascular complications [37, 39, 41]. Similarly, the strong correlation between high salt consumption and elevated blood pressure further supports the existing body of evidence that emphasizes dietary interventions as a critical component of hypertension management [42, 43].

Identifying individuals with central obesity, or high salt and oily food consumption enables targeted lifestyle counseling and community-based interventions aimed at reducing hypertension risk [44–46]. Furthermore, the inclusion of non-modifiable factors such as age and family history allows for more personalized risk stratification and resource allocation in screening programs [47]. The use of these variables, which are easily measurable at the primary care level, ensures that the model can be implemented in routine NCD screening settings across Indonesia, ultimately supporting more efficient and equitable hypertension prevention efforts.

## Limitation and Future Direction

Our study has several limitations that should be acknowledged. First, the cross-sectional design prevents the inference of causality and introduces a risk of reverse causality with lifestyle factors. Second, the dataset has notable limitations in scope and quality; it is primarily sourced from non-hospital settings, which may limit applicability to patients with more complex conditions, and it lacks clinical variables such as medication status. Furthermore, the exclusion of a large volume of records due to significant data quality challenges may limit the representativeness of our sample and its generalizability across Indonesia’s diverse demographic groups.

These limitations highlight several key directions for future work. A critical next step is to conduct a longitudinal study to establish causal relationships and move beyond the associations identified here. To address data gaps and improve model performance, future research should focus on integrating richer datasets to improve specificity, capture important variables like medication status, other physiological systems like renal function, or incorporating other outcome categories such as prehypertension [48–50]. Concurrently, expanding data collection to rural and under-represented populations is essential for enhancing the model’s generalizability and ensuring its equitable application [18, 37]. Finally, further model enhancement could be achieved by incorporating novel data streams from wearables and genomics [48, 51], while the ultimate goal remains the practical implementation of this tool within the national SATUSEHAT platform to create a scalable public health solution for hypertension screening [52, 53].

## Conclusion

In conclusion, this study highlights the potential of machine learning models to enhance personalized hypertension prediction in Indonesia. While the inclusion of personal hypertension history improves predictive accuracy, the models demonstrate robustness even when such data is unavailable. Out of 5 algorithms used, XGBoost and LightGBM consistently achieved the highest AUC values in both models. This finding highlights the effectiveness of ensemble methods, particularly XGBoost and LightGBM, for hypertension risk prediction. Considering that LightGBM has a low memory usage, this algorithm demonstrates better performance compared to XGBoost in terms of handling large scales of data. Top contributing factors that were found through SHAP analysis are age, family history of hypertension, body weight, waist circumference, and salt consumption. Model B offers a valuable and generalizable approach for broader risk screening, particularly where patient history may be unavailable or unreliable, while also providing insights into key modifiable and non-modifiable predictors of hypertension.

The model provides a risk score for classifying an individual as likely hypertensive, rather than predicting specific systolic or diastolic blood pressure measurements. The proposed integration of these models into existing platforms such as the SATUSEHAT Mobile app could represent a practical application of AI in public health, offering a scalable solution for hypertension screening. However, further research is needed to improve specificity, validate the models in diverse populations, and explore the long-term impact of AI-driven hypertension management strategies.

## Supplementary information

Below is the link to the electronic supplementary material.

Supplementary file1 (DOCX 120 KB)

## Data Availability

No datasets were generated or analysed during the current study.
