# Supplementary material for: Prediction of Personalised Hypertension Using Machine Learning in Indonesian Population
Source: J Med Syst. 2025 Oct 13;49(1):137. doi: 10.1007/s10916-025-02253-5 (PMC12515743; doi:10.1007/s10916-025-02253-5)
Supplement: Supplementary file 1 — Supplementary file1 (DOCX 120 KB) [file 10916_2025_2253_MOESM1_ESM.docx]

**Appendix 1. Model Framework**


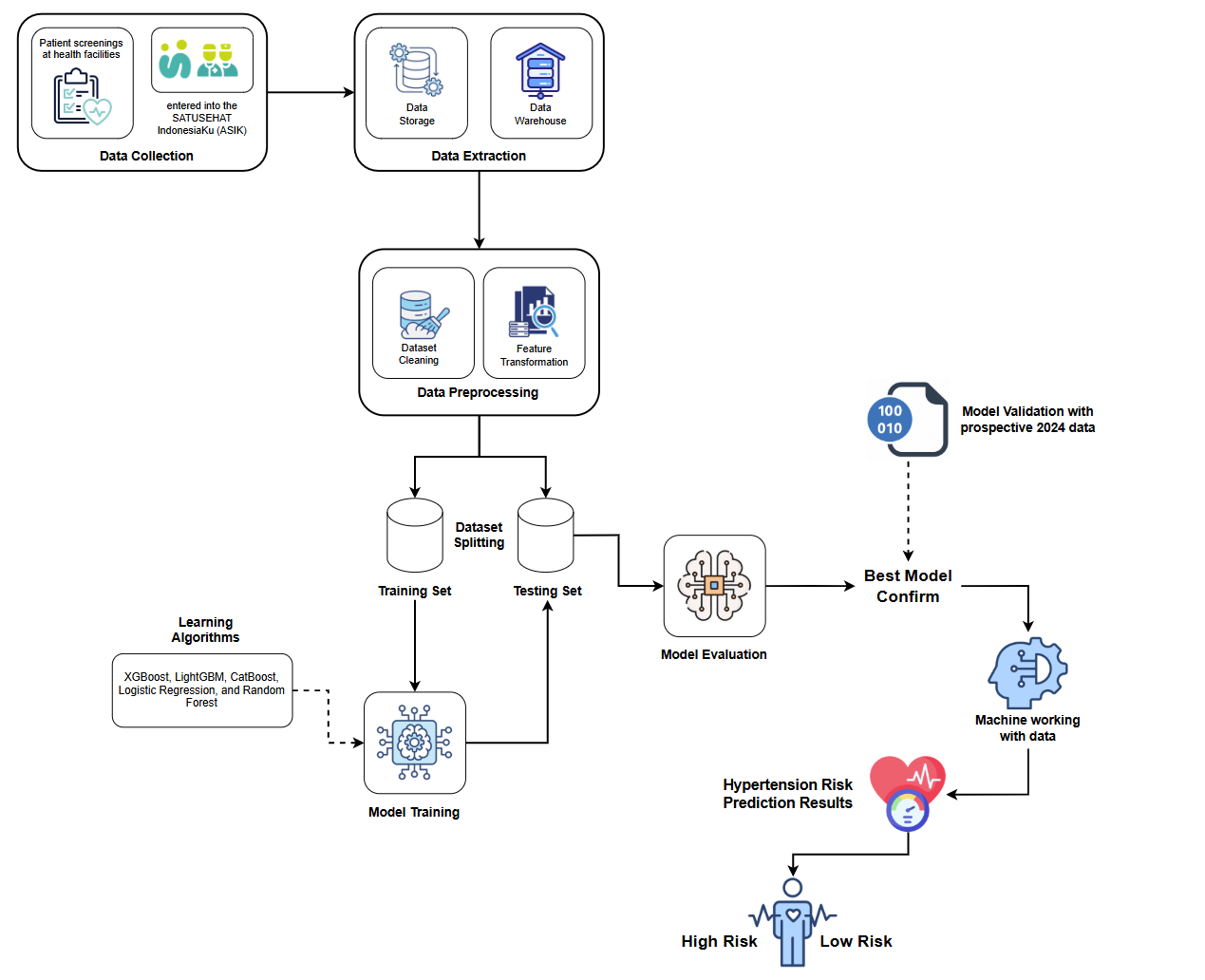


**Appendix 2. Model Interpretability using SHAP**

The SHAP explanation model can be represented as:


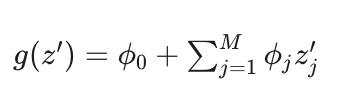


where g is the explanation model, z′ is the coalition vector (a simplified representation of features being present or absent), M is the maximum coalition size, and ϕj​ is the Shapley value for feature j, representing its contribution.
